# Somatostatin receptor saturation after administration of high peptide amounts of [^177^Lu]Lu-HA-DOTATATE: when enough is enough

**DOI:** 10.1186/s13550-022-00946-3

**Published:** 2022-12-14

**Authors:** Hinke Siebinga, Chayenne H. A. M. Veerman, Linda de Wit-van der Veen, Marcel P. M. Stokkel, Jeroen J. M. A. Hendrikx, Else A. Aalbersberg

**Affiliations:** 1 Department of Pharmacy and Pharmacology, The Netherlands Cancer Institute, Plesmanlaan 121, 1066 CX Amsterdam, The Netherlands; 2 Department of Nuclear Medicine, The Netherlands Cancer Institute, Plesmanlaan 121, 1066 CX Amsterdam, The Netherlands

**Keywords:** PRRT, [^177^Lu]Lu-HA-DOTATATE, Receptor saturation, Peptide amount, Mass dose

## Abstract

**Background:**

Receptor saturation during peptide receptor radionuclide therapy (PRRT) could result in altered [^177^Lu]Lu-HA-DOTATATE uptake in tumors and organs. Therefore, receptor expression status and effects of different (unlabeled) administered peptide amounts during PRRT need to be evaluated. The aim of this study was to assess potential receptor saturation during PRRT by comparing organ and tumor uptake after administration of [^177^Lu]Lu-HA-DOTATATE with low, standard and high administered peptide amounts in patients with advanced metastatic neuroendocrine tumors (NETs).

**Methods:**

Data of NET patients that received 7.4 GBq 177-Lutetium labeled to a low or high amount of HA-DOTATATE were retrospectively included. From included patients other PRRT cycles, containing standard administered peptide amounts, were included for intra-patient comparison. Uptake quantification was performed for spleen, liver, kidney, bone marrow, blood pool and tumor lesions on post-treatment SPECT/CT scans. A paired Wilcoxon signed-rank test was performed to determine uptake differences between two adjacent cycles for each patient.

**Results:**

Thirteen patients received [^177^Lu]Lu-HA-DOTATATE with a high administered peptide amount (mean 346 µg vs 178 µg standard peptide amount). Low peptide amounts were administered to fifteen patients (mean 109 µg vs 202 µg standard peptide amount). High administered peptide amount resulted in significantly lower [^177^Lu]Lu-HA-DOTATATE uptake in the spleen (*p* = 0.00012), kidney (*p* = 0.013) and tumor lesions (*p* < 0.0001) versus standard peptide amounts. For low administered peptide amount, uptake was increased in the spleen (*p* = 0.015), while tumor uptake was significantly reduced (*p* = 0.015) compared to uptake after administration of standard peptide amounts.

**Conclusions:**

These findings confirmed a peptide amount-dependent organ and tumor accumulation for [^177^Lu]Lu-HA-DOTATATE, with receptor saturation in spleen for high and standard peptide amounts, while tumor and kidney receptor saturation occur only with high administered peptide amounts. A high peptide amount (~ 350 µg) is not recommended for standard-dose PRRT and standard amounts (~ 200 µg) seem more suitable to achieve optimal tumor accumulation with limited organ uptake.

**Supplementary Information:**

The online version contains supplementary material available at 10.1186/s13550-022-00946-3.

## Introduction

Somatostatin receptors (SSTRs) are expressed throughout the body in different organs, such as adrenal gland, spleen, liver, pancreas and kidney [1]. Most neuroendocrine tumors (NETs) show an overexpression of SSTRs, which is used as a target for both diagnosis and therapy [2, 3]. While the standard radionuclide treatment of four cycles of 7.4 GBq of Lutetium-177 DOTATATE ([^177^Lu]Lu-DOTATATE) (180–300 µg) has proven to be safe and effective, not all patients benefit and there is still a lot of debate on ways to improve efficacy. Effectiveness of radiolabeled somatostatin analogues (SSAs) is related to tumor SSTR occupancy and although an overexpression in tumors compared to healthy tissues is present [4–6], exact in vivo receptor expression levels remain unknown. Knowledge regarding SSTR expression concentrations in tissues and tumors would be highly relevant as it can help to make PRRT more effective and less toxic.

Purposefully saturating receptors could be a relevant approach to potentially decrease organ uptake and related toxicity. Higher unlabeled peptide amounts could occupy SSTRs, resulting in reduced uptake in organs due to less possibility for radiolabeled SSAs to bind and subsequently internalize into those tissues. However, receptor saturation in tumors might limit maximum achievable tumor uptake and thus result in reduced therapeutic radiation doses. For application of such an approach, receptor expression status and effects of different administered amounts of radiolabeled SSAs need to be evaluated. Amount-dependent differences in radiolabeled SSA uptake, possibly caused by receptor saturation, were previously observed in several preclinical experiments [7–9]. Although results in patients are still limited, Sabet et al. preliminary showed a limited receptor capacity in normal tissues (liver and spleen) but no receptor saturation in tumors, which was assessed by comparing uptake on Gallium-68 DOTATOC ([^68^Ga]Ga-DOTATOC) PET/CT before and directly after standard-dose PRRT with [^177^Lu]Lu-DOTATATE [10]. Given the fact that tumor SSTRs were not saturated, one could argue that administration of higher activities is feasible to enhance effectivity and cold-SSAs prior to PRRT could limit normal tissue accumulation and reduce toxicity. In such cases, physiological processes can be saturated to some extent without evident effects on tumor accumulation. First evidence for this phenomenon is provided in the studies by Aalbersberg et al. [11] and Velikyan et al. [12]. Both studies show that co-administration of unlabeled SSA has a limited positive effect on the uptake in target lesions, but can decrease accumulation in the thyroid, spleen, liver, and kidneys. In a recent study by Jahn et al*.*, static and dynamic PET-imaging was performed up to 7 h after 400 µg octreotide plus [^68^Ga]Ga-DOTATOC (~ 167 MBq and ~ 24 µg) in patients with proven NET [13]. Based on these data, it can be hypothesized that unlabeled peptide (or co-administration of SSAs prior to PRRT) could have a positive effect on the therapeutic balance between anti-tumor effect and toxicity. Subsequently, improper selection of SSA peptide dosages could result in less effective anti-tumor activity.

Currently, there is limited evidence regarding total peptide amounts that should be used for labeling procedures, and the current guidelines are mainly based on consensus in the field [14]. There is an urgent need for a better understanding of the effect of varying (unlabeled) administered peptide amounts on [^177^Lu]Lu-DOTATATE uptake during PRRT. This could help to eventually optimize efficacy and toxicity, by potentially reducing organ uptake without affecting, or even increasing, tumor accumulation. Therefore, the aim of this study was to assess receptor saturation during PRRT by comparing organ and tumor uptake after administration of [^177^Lu]Lu-HA-DOTATATE with low, standard and high peptide amounts at standard-dose 7.4 GBq in patients with advanced metastatic NETs.

## Methods

### Patients

This retrospective data analysis was approved by the Institutional Review Board (IRBd21-187) of the Netherlands Cancer Institute (Amsterdam, The Netherlands). The PRRT protocol included four cycles of 7.4 GBq [^177^Lu]Lu-HA-DOTATATE with a 10-week interval between cycles. Patients receiving reduced activity doses (3.7 or 5.6 GBq) were excluded from analysis.

Patients who received ^177^Lu-labeled to a high amount (~ 350 µg total peptide per administration) of HA-DOTATATE (i.e., low specific activity) were retrospectively included. Furthermore, patients who received a low amount (~ 100 µg total peptide per administration) of HA-DOTATATE (i.e., high specific activity) labeled to ^177^Lu were included for a second analysis. From all included patients the other PRRT cycles, containing standard administered peptide amounts (~ 200 µg total peptide per administration), were included for intra-patient comparison.

### [^177^Lu]Lu-HA-DOTATATE preparation

[^177^Lu]Lu-DOTA-iodo-Tyr^3^-octreotate (HA-DOTATATE; HA, high-affinity) was prepared as previously described, where 10 GBq ^177^Lu-chloride solution ([^177^Lu]LuCl_3_) is labeled to 250 µg HA-DOTATATE peptide [15]. In case of low specific activity of the [^177^Lu]LuCl_3_ (i.e., > 25 µg total lutetium mass, in case of production via the carrier added route), the amount of HA-DOTATATE peptide was increased up to 500 µg to ensure adequate binding of ^177^Lu to the peptide. This resulted in higher total peptide amounts per administration and thus lower specific activities at standard 7.4 GBq dose PRRT.

Furthermore, in August 2021 labeling procedures were adjusted to 18 GBq (produced via no-carrier added route) preparations to accommodate multiple patient dosages, while the amount of HA-DOTATATE peptide that was added to [^177^Lu]LuCl_3_ for radiolabeling remained 250 µg. Accordingly, lower total peptide amounts were administered to patients resulting in higher specific activities of [^177^Lu]Lu-HA-DOTATATE at standard 7.4 GBq dose PRRT.

### Image analysis

Single-photon emission computerized tomography (SPECT)/CT scans of thorax abdomen were acquired ~ 24-h post-injection, performed on a Symbia T2 (Siemens GmbH, Erlangen, Germany) equipped with a medium energy collimator. Two protocols were used: (1) energy window 208 keV (± 10%) with 20% lower scatter, 64 views, 14 s/view or (2) energy window 208 keV (± 10%) with 10% lower scatter and a general scatter window from 18.5 to 166 keV, 96 views, 13 s/view. For each protocol, a calibration factor (counts/MBq) for the clinical SPECT reconstructions was determined using a ^177^Lu-filled NEMA Image Quality phantom. Absolute activity concentrations (kBq/mL) in organs were determined by placing spherical volumes-of-interest (VOIs) in the spleen, liver and kidney cortex (all diameter 30 mm). Multiple regions-of-interest (ROIs, size to fit) were placed in the corpus of L2-L4 to estimate bone marrow uptake, and blood pool activity was estimated by placing ROIs in the descending aorta in four consecutive slices (size to fit). Activity concentrations in tumor lesions with a diameter > 2 cm were determined by placing spherical VOIs (diameter 20 mm, located in the highest uptake region). All segmented tumor lesions were included separately for analysis.

### Statistical evaluation

All statistical analyses were performed in R (version 4.1.3) [16]. Patient characteristics between groups were compared with a Wilcoxon signed-rank test. Organ and tumor activity concentrations of all cycles were compared between cycles to identify uptake trends over cycles and specific errors or outliers. In addition, intra-patient uptake differences for low versus standard peptide amounts and for standard versus high peptide amounts were analyzed for both organs and tumors using paired Wilcoxon signed-rank tests. For this, per patient two adjacent cycles with standard and low/high administered peptide amount were included. A *p*-value less than 0.05 was considered statistically significant.

## Results

**Fig. 1:**
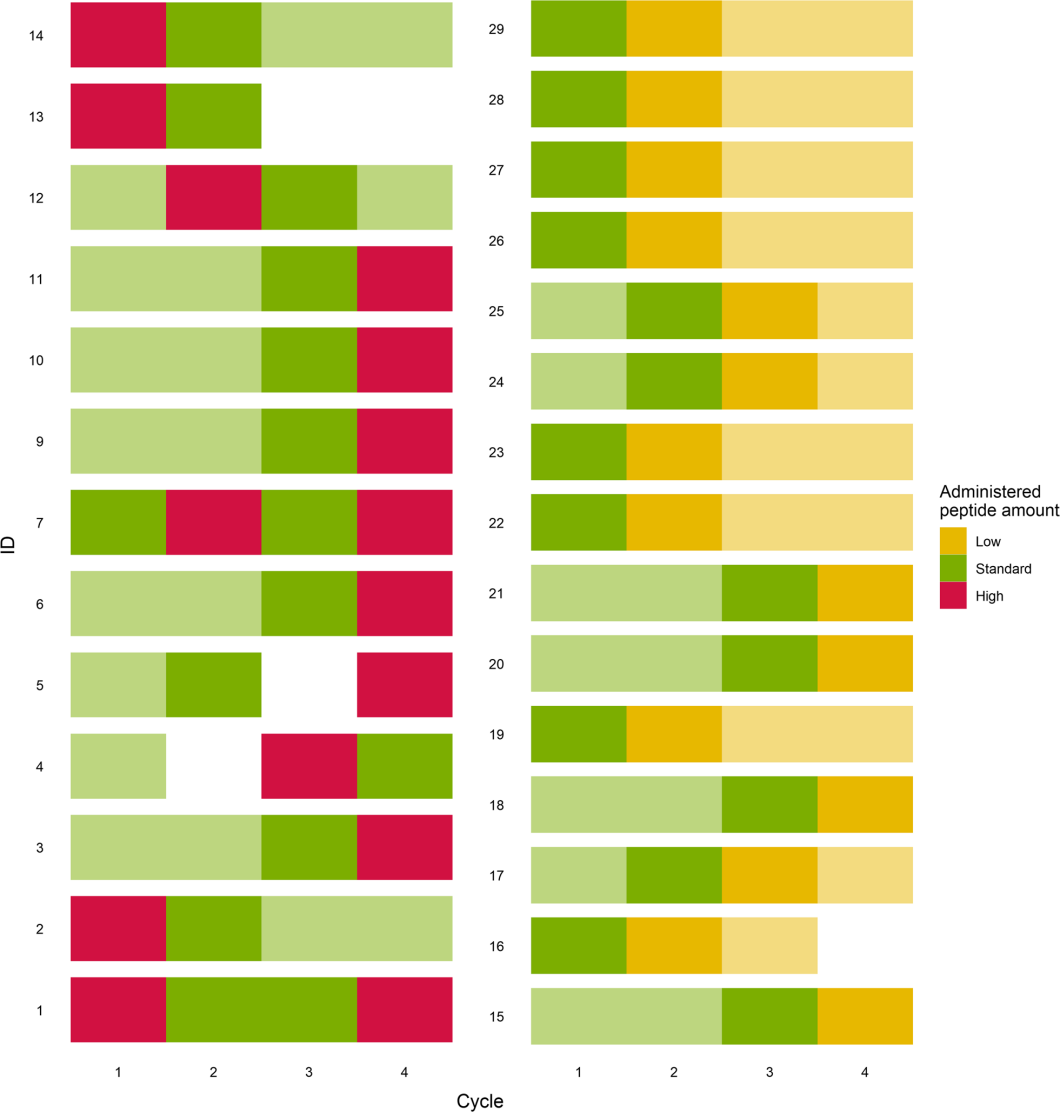
Overview of low, standard or high administered peptide amount of [^177^Lu]Lu-HA-DOTATATE per cycle for each patient, where dark colored blocks represent included cycles for paired Wilcoxon analysis

A total of 28 patients were selected that received at least one [^177^Lu]Lu-HA-DOTATATE cycle with standard peptide amounts and at least one cycle with either low or high administered peptide amounts. Patient characteristics are shown in Tables 1 and 2, representing patients receiving a high and low administered peptide amount, respectively. Comparison of uptake between both standard groups showed no significant differences for spleen, liver, kidney and bone marrow uptake (*p* = 0.187, *p* = 0.209, *p* = 0.239 and *p* = 0.781, respectively), and therefore, both standard groups represent typical PRRT patients. Information regarding the cycles included for both analyses is provided in Fig. 1.

**Table 1:** Patient characteristics of patients receiving a PRRT cycle with a high administered peptide amount

*N*	13*
Male (%)	7 (54%)
Age (years)	66 ± 8
Weight (kg)	77.1 ± 22.2

	High peptide amount	Standard cycle	*p*-value
Administered peptide amount (µg)	346 ± 32.8	178 ± 8.84	< 0.0001
Injected radioactivity (MBq)	7492 ± 109.7	7464 ± 86.03	0.303
Specific activity (MBq/µg)	21.9 ± 2.52	42.1 ± 2.14	< 0.0001

^*^Of which 2 patients were included twice for paired Wilcoxon analysis

Continuous variables are shown as mean ± standard deviation and categorical variables as number (%)

**Table 2:** Patient characteristics of patients receiving a PRRT cycle with a low administered peptide amount

*N*	15
Male (%)	7 (47%)
Age (years)	68 ± 6
Weight (kg)	76.7 ± 14.2

	Low peptide amount	Standard cycle	*p*-value
Administered peptide amount (µg)	109 ± 6.60	202 ± 14.7	< 0.0001
Injected radioactivity (MBq)	7300 ± 274.0	7480 ± 133.3	0.0554
Specific activity (MBq/µg)	67.3 ± 5.26	37.3 ± 2.88	< 0.0001

Continuous variables are shown as mean ± standard deviation and categorical variables as number (%)

### High peptide amounts

[^177^Lu]Lu-HA-DOTATATE with high peptide amounts was administered to thirteen patients, of which two patients received a dose with a high peptide amount twice. This resulted in the inclusion of data from 30 cycles (fifteen high and fifteen standard peptide amounts) for paired analysis. From these selected patients, 29 tumor lesions were included for tumor analysis. Mean (± SD) administered high peptide amount was 346 µg (± 32.8 µg) versus 178 µg (± 8.84 µg) standard peptide amount (mean specific activity 21.9 MBq/µg versus 42.1 MBq/µg, respectively).

**Fig. 2:**
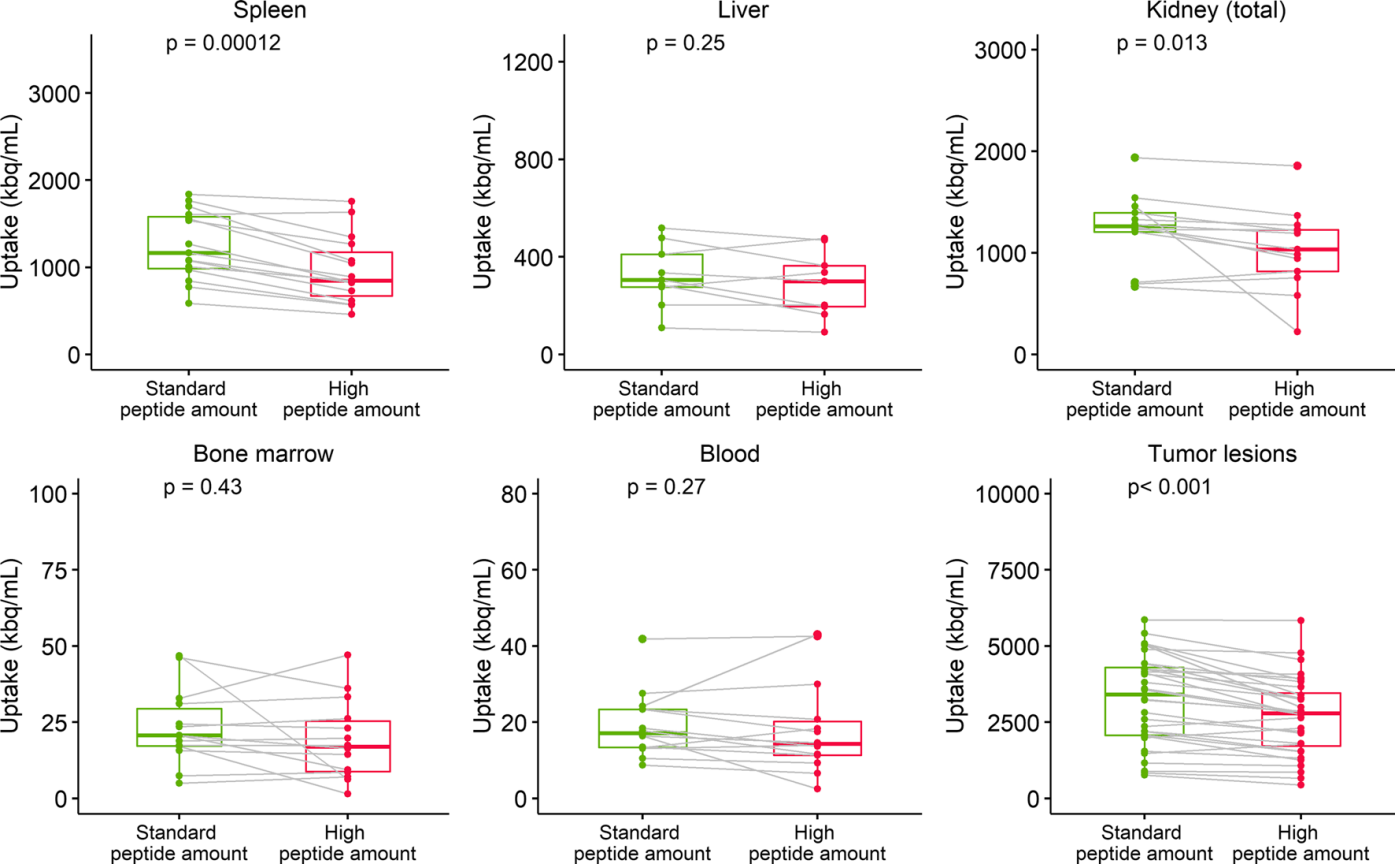
Results from paired Wilcoxon analyses of organ and tumor uptake differences between standard and high administered peptide amount of [^177^Lu]Lu-HA-DOTATATE

**Fig. 3:**
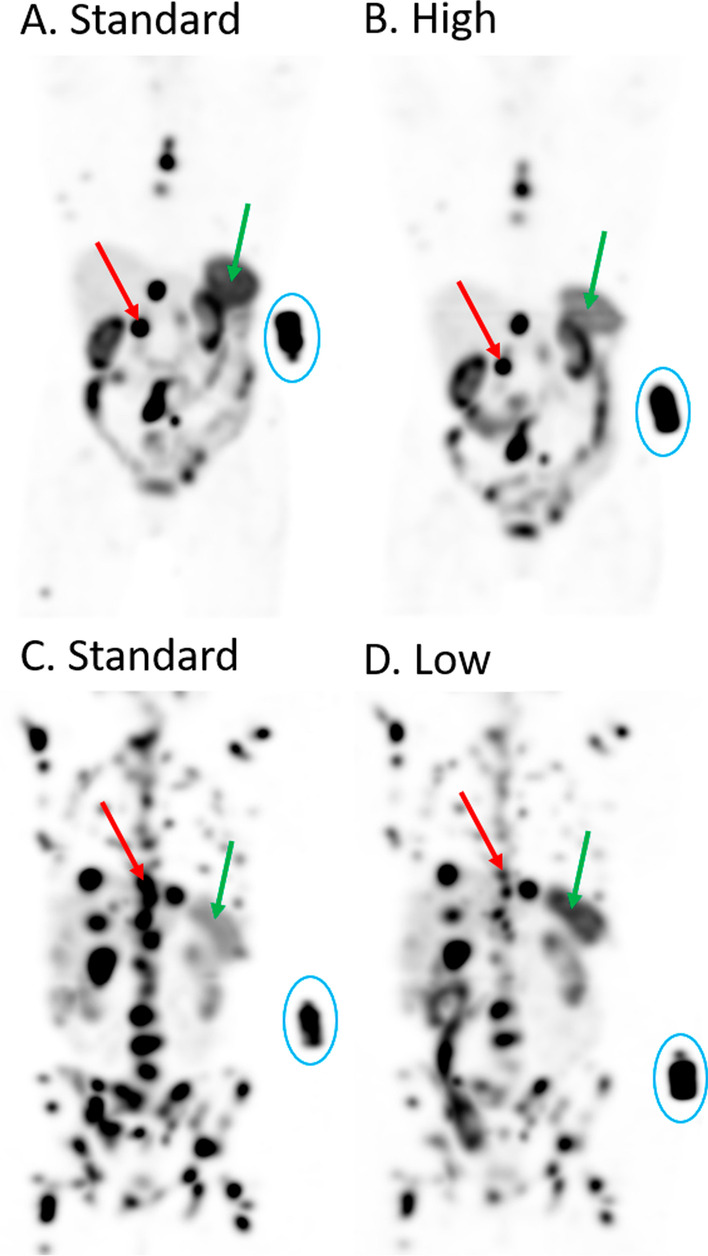
Maximum intensity projections of SPECT images acquired 24 h after injection of [^177^Lu]Lu-HA-DOTATATE. (**A**, **B**) Patient with both standard (cycle 2) and high (cycle 1) peptide amount. (**C**, **D**) Patient with both standard (cycle 1) and low (cycle 2) peptide amount. Green arrows indicate the spleen, which shows decreased uptake with high peptide amount and increased uptake with low peptide amount. Red arrows point to tumor lesions, which show decreased uptake in both high and low peptide amounts. A reference standard with known amount of radioactivity is scanned with each patient (encircled in blue)

Trends in tumor and organ uptake over all cycles are depicted in Additional file 1: Figure S1. Administration of high versus standard peptide amounts resulted in significantly decreased [^177^Lu]Lu-HA-DOTATATE uptake in the spleen (mean uptake 965.3 vs 1252 kBq/mL, *p* = 0.00012), kidney (mean uptake 1036 vs 1227 kBq/mL, *p* = 0.013) and tumor lesions (mean uptake 2700 vs 3234 kBq/mL, *p* < 0.0001). For liver, bone marrow and blood pool no significant differences were observed. Results of this paired analysis are shown in Fig. 2. An example of a patient with both standard and high peptide amounts is shown in Fig. 3A–B.

### Low peptide amounts

Low peptide amounts were administered to fifteen patients, which resulted in the inclusion of data from 30 cycles for paired analysis (fifteen low and fifteen standard peptide amounts). From these fifteen patients, a total of 36 tumor lesions were included for tumor analysis. Mean (± SD) low administered peptide amount was 109 µg (± 6.60 µg) versus 202 µg (± 14.7 µg) standard peptide amount (mean specific activity 67.3 MBq/µg versus 37.3 MBq/µg, respectively).

**Fig. 4:**
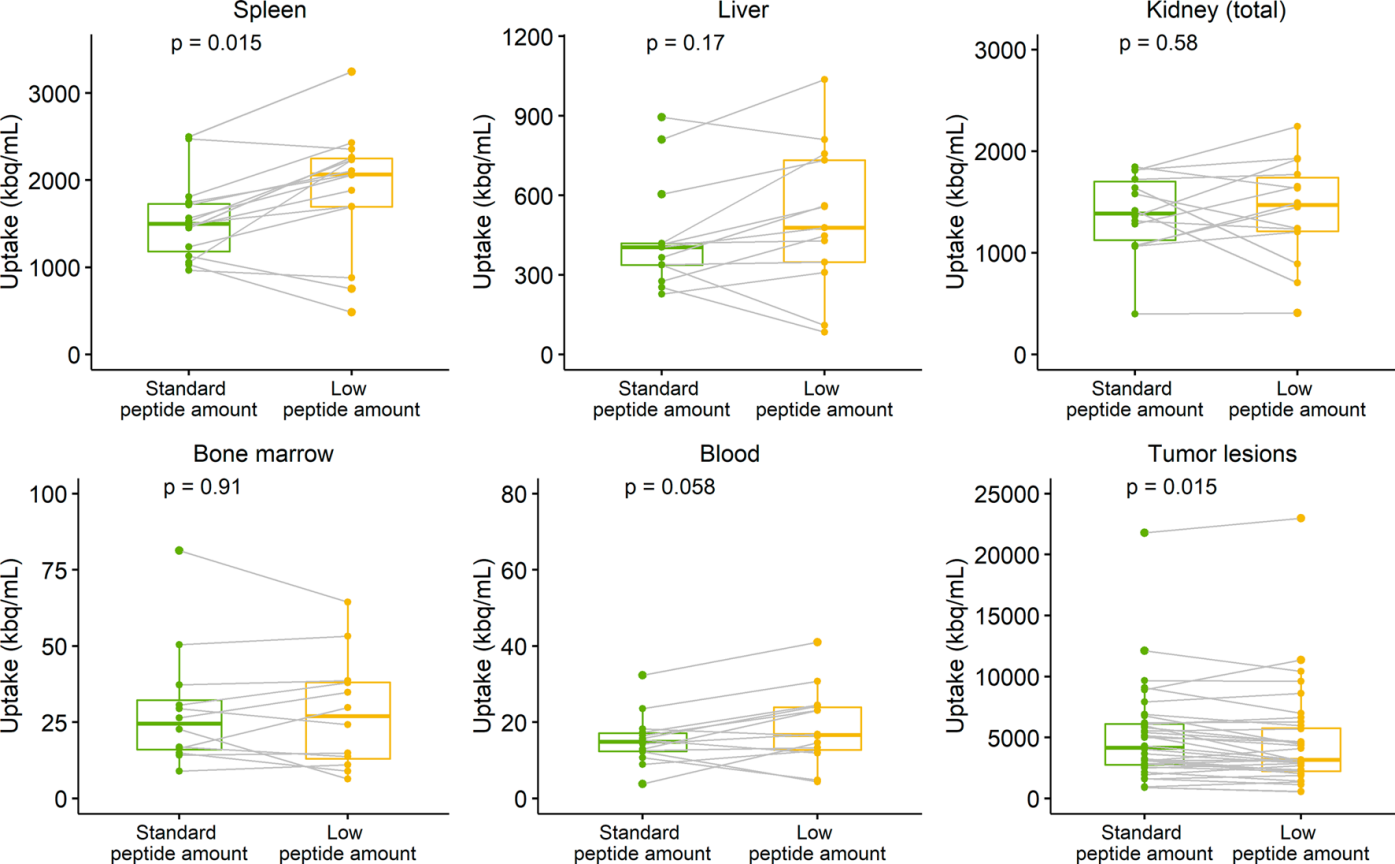
Results from paired Wilcoxon analyses of organ and tumor uptake differences between standard and low administered peptide amounts activity of [^177^Lu]Lu-HA-DOTATATE

Overall uptake trends in organs and tumors over all cycles are shown in Additional file 1: Figure S2. Administration of low versus standard peptide amounts resulted in significantly increased [^177^Lu]Lu-HA-DOTATATE uptake in the spleen (mean uptake 2068 vs 1498 kBq/mL, *p* = 0.015) and reduced uptake in tumor lesions (mean uptake 3176 vs 4174 kBq/mL, *p* = 0.015). For kidney, liver, bone marrow and blood pool no significant differences were observed. Results of this paired analysis are shown in Fig. 4. An example of a patient with both standard and low peptide amounts is shown in Fig. 3C–D.

## Discussion

This study assessed normal organ and tumor accumulation during PRRT after administration of low, standard and high peptide amounts of [^177^Lu]Lu-HA-DOTATATE to provide evidence of possible receptor saturation. Results showed a significant decrease for [^177^Lu]Lu-HA-DOTATATE uptake in spleen, kidney and tumor lesions of high versus standard peptide amounts. Likewise, administration of low peptide amount resulted in an evident increase of [^177^Lu]Lu-HA-DOTATATE uptake in spleen, while tumor accumulation was significantly decreased compared to standard administered peptide amounts. These results imply occurrence of organ saturation in the spleen during PRRT even at standard peptide amounts (~ 200 µg). For the kidneys, receptor saturation occurs only at high peptide amounts, since no uptake differences were observed for low versus standard administered peptide amounts. Tumor uptake was also saturated at higher peptide amounts, but more interestingly, administration of a low total peptide amount resulted in a significant reduction in tumor uptake. This amount-dependent tumor accumulation is probably caused by competitive uptake in the spleen, which acts as a so-called sink organ. All in all, this study confirms the need for standardized peptide dosing to achieve an optimal and reproducible tumor uptake with minimal negative effects of receptor saturation or normal tissue competition. Based on these results, standard peptide amounts (~ 200 µg) are recommended because of optimal tumor uptake, while spleen uptake was reduced compared to low administered peptide amounts due to saturation. Such total peptide amounts are comparable to amounts administered with Lutathera® and as recommended by guidelines (total peptide amount of 100–200 µg, not exceeding 250 µg per patient dose) [14, 17].

Some preclinical studies have assessed saturation status or optimal peptide amounts in mice with administration of radiolabeled SSAs [7–9]. Also for patients with NETs, some pilot studies examined the impact of peptide masses on radiolabeled SSA uptake in organs and tumors [12, 18]. All these studies showed that uptake in SSTR-positive tissues and/or tumors is to some extent dependent on injected peptide amount. While those studies did not focus on assessing receptor saturation, Sabet et al*.* aimed to identify receptor saturation during standard-dose (7.4 GBq; 54 GBq/µmol) PRRT by performing SSA-based PET imaging before and immediately after PRRT [10]. Similar to our results, limited receptor capacity of the spleen was described in this small retrospective study (*n* = 5). Results of decreasing liver uptake after PRRT were not in accordance with our findings. This might be caused by the limited data for normal liver uptake, since many NET patients suffer from extensive liver metastases which hampers healthy liver quantification. Furthermore, Sabet et al*.* did not report any relevant saturation of SSTRs in target tumor lesions after their standard-dose PRRT. However, our data did imply saturated uptake in tumors after administration of high peptide amounts. In accordance with this finding, Velikyan et al*.* [12] also demonstrated tumor saturation when performing SSA-based imaging after co-administration of 250–500 µg unlabeled octreotide (short-acting SSA). Therefore, caution is probably warranted when increasing injected ^177^Lu-activities, because total peptide amounts may increase accordingly. A standard protocol of 7.4 GBq PRRT with a high total peptide amount is no longer administered in our hospital.

As with any retrospective study, there are some limitations on the methodology and patient selection process. Consequently, bias was introduced to our analyses and, thus, all possible sources of bias are addressed below. Still, it remains important to share these retrospective data, especially since a prospective study with different administered peptide amounts is not likely to be performed. A paired Wilcoxon analysis was performed, where accumulation was compared between low/high versus standard administered peptide amounts in the same patient. An important advantage of such a paired analysis is that inter-patient differences, such as tumor load, renal function or clinical status, will have a limited impact on the outcomes. Still, uptake profiles could have varied between two sequential cycles because of radiation effects. For tumor uptake, a decreased accumulation would be expected in later cycles, as was previously described [19]. This could have played a role in our analysis, because in all patients receiving low administered peptide amounts, the standard cycle was prior to lower one (see Fig. 1). If tumor accumulation depends on peptide amount, an increased uptake after administration of low peptide amounts compared to standard dosing would be expected, which is contrary to the ‘cycle effect’ as later cycles would result in a reduced tumor uptake. Interestingly, indeed a reduced tumor uptake was observed for low versus standard administered peptide amount, which could have been caused by this cycle effect. However, in general no visual trends in decreased uptake over cycles were observed for tumors (see Additional file 1: Figure S2). As mentioned previously, a physiological explanation for decreased tumor uptake in this group would be the elevated activity level in the spleen resulting in decreased total available [^177^Lu]Lu-HA-DOTATATE for tumor uptake.

For our analysis regarding high administered peptide amounts, an additional analysis only including patients with the high peptide amount cycle prior to a standard dosing cycle (*n* = 6) still resulted in a significant decrease in tumor uptake (*p* = 0.011) for high peptide amount, which is contrary to the expected effect over cycles. This confirms receptor saturation in target tumors of advanced NET patients that received high total peptide amounts. For organs, no apparent evidence for decreased organ uptake caused by radiation effects was published previously. This was also not observed while looking at uptake trends over cycles for all patients (see Additional file 1: Figures S1 and S2). Therefore, uptake differences caused by comparison of two different cycles will probably not have impacted our findings. Another limitation for this analysis regarding patients receiving high peptide amounts was that two patients were included twice, which may bias results. However, additional analyses including only one high peptide amount cycle from these patients did not alter results for organ and tumor uptake (data not shown).

All patients in the high administered peptide amount group discontinued long-acting SSAs 4 weeks prior to PRRT when applicable. However, in the low administered peptide amount group, SSAs were not discontinued in one cycle, while this was the case in the adjacent cycle for three patients. For two of those patients (ID 24 and ID 25) this might have biased results, since the SSA was discontinued in the low peptide amount cycle and this could lead to an increased organ uptake compared to the prior cycle (similar to the low peptide amount effect). However, looking at trends in organ uptake (see Figure S2) it is not expected that this intra-patient difference in discontinuation of SSAs impacted our conclusions.

Uptake in organs and tumors was only measured as absolute radioactivity at 24-h post-injection using SPECT/CT imaging. Unfortunately, uptake at one time point cannot directly be transferred into absorbed radiation doses. For this, time–activity curves are needed that describe both the initial (maximal) uptake and excretion phase, as both have an equal important contribution to the absorbed radiation dose. Still, the initial activity concentration is descriptive for accumulation, and we believe this approach is suitable for the evaluation of receptor saturation after administration of [^177^Lu]Lu-HA-DOTATATE. Further research could focus on effects of fluctuating administered peptide amounts on absorbed doses to organs at risk and tumor lesions.

## Conclusions

Retrospective data analyses were performed to assess receptor saturation during PRRT with low, standard and high administered peptide amounts of [^177^Lu]Lu-HA-DOTATATE. High administered peptide amounts resulted in a significant reduced uptake in spleen, kidney and tumor lesions for all patients. A low administered peptide amount evidently increased spleen uptake, while tumor uptake was reduced. These findings confirm a peptide amount-dependent saturation of organ and tumor accumulation for [^177^Lu]Lu-HA-DOTATATE, in which receptor saturation in the spleen is achieved at high and standard administered peptide amounts, while tumor and kidney receptor saturation only occur after administration of high peptide amounts. In this respect, it can be concluded that a high administered peptide amount is not recommended for PRRT, confirming the expert opinion in the EANM guideline with real-world data.

## Supplementary Information

**Additional file 1: Figures S1 and S2.** Trends in [^177^Lu]Lu-HA-DOTATATE organ and tumor uptake over cycles per patient.

## Data Availability

The datasets analyzed during the current study are available from the corresponding author on reasonable request.
