# Supplementary material for: Somatostatin receptor saturation after administration of high peptide amounts of [177Lu]Lu-HA-DOTATATE: when enough is enough
Source: EJNMMI Res. 2022 Dec 14;12:74. doi: 10.1186/s13550-022-00946-3 (PMC9751249; doi:10.1186/s13550-022-00946-3)
Supplement: Supplementary file 1 — Additional file 1: Figures S1 and S2. Trends in [177Lu]Lu-HA-DOTATATE organ and tumor uptake over cycles per patient. [file 13550_2022_946_MOESM1_ESM.pdf]

Supplementary Information

Somatostatin Receptor Saturation after Administration of High Peptide Amounts of [^{177}Lu]Lu-HA-DOTATATE – when enough is enough

H. Siebinga[1,2], C.H.A.M. Veerman[2], B.J. de Wit – van der Veen[2], M.P.M. Stokkel[2], J.J.M.A. Hendrikx[1,2], E.A. Aalbersberg[2]

1 Department of Pharmacy & Pharmacology, The Netherlands Cancer Institute, Amsterdam, The Netherlands.

2 Department of Nuclear Medicine, The Netherlands Cancer Institute, Amsterdam, The Netherlands.

Corresponding author: e.aalbersberg@nki.nl, Plesmanlaan 121, 1066 CX Amsterdam, The Netherlands

Supplemental Figure 1 – Trends in [^{177}Lu]Lu-HA-DOTATATE organ and tumor uptake over cycles per patient, where red dots represent cycles with a high administered peptide amount.

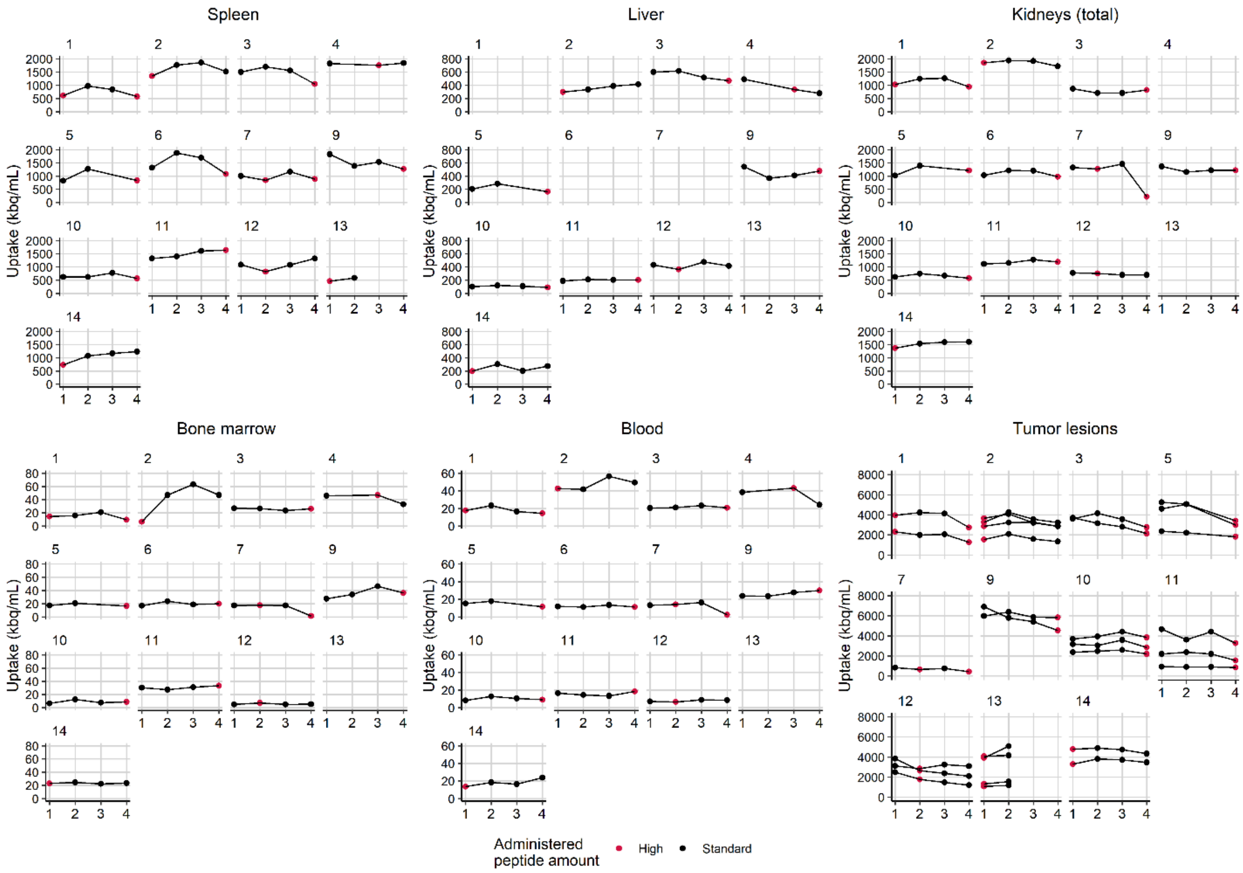

Supplemental Figure 2 Trends in [^{177}Lu]Lu-HA-DOTATATE organ and tumor uptake over cycles per patient, where yellow dots represent cycles with a low administered peptide amount.

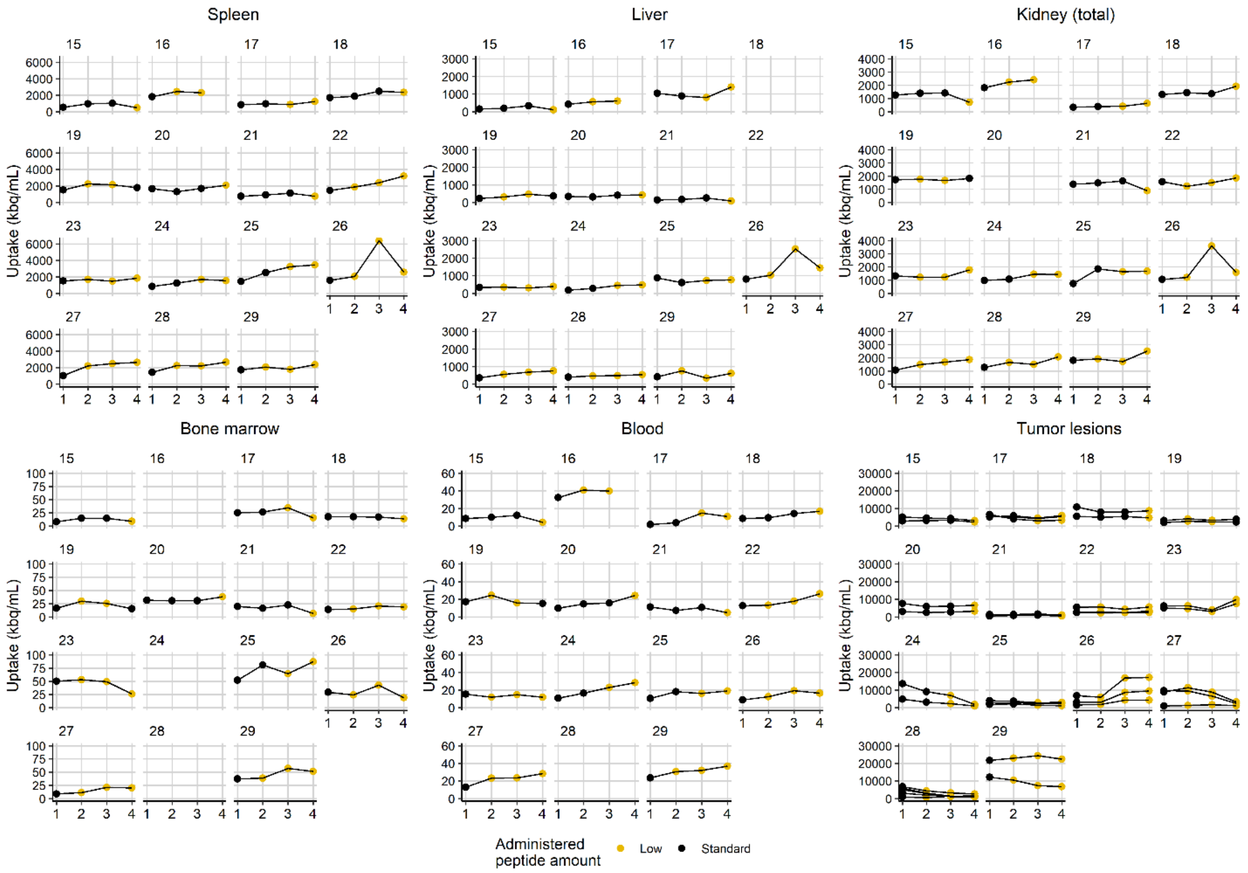

Missing data due to diffuse liver metastases (n=2), diffuse bone metastases or spill-out by nearby tumor into the bone marrow (n=3), spill-out into the aorta by nearby tumor lesion (n=1), or no average being available due to a solitary kidney (n=1).
